# The Correlation of Smartphone Screen Time With Resting Respiratory Rate, Head-Neck Alignment, and Chest Expansion Among University Students

**DOI:** 10.7759/cureus.114396

**Published:** 2026-08-12

**Authors:** Vaishnavi S Tapale, Radhika Chintamani

**Affiliations:** 1 Department of Physiotherapy, Krishna Vishwa Vidyapeeth (Deemed to be University), Karad, IND; 2 Department of Orthopaedic Manual Therapy, Krishna Vishwa Vidyapeeth (Deemed to be University), Karad, IND

**Keywords:** craniovertebral angle, forward head posture, head-neck alignment, posture, respiratory rate, smartphone screen time

## Abstract

Background and objective

Smartphone usage has increased considerably among young adults and is often associated with prolonged neck flexion and forward head posture. Excessive screen time may lead to alterations in head-neck alignment, musculoskeletal adaptations, and changes in respiratory mechanics. Previous studies have suggested that poor posture can influence breathing patterns and respiratory function; however, evidence regarding the relationship between smartphone screen time, head-neck alignment, and resting respiratory rate remains limited. This study aimed to assess the correlation between smartphone screen time, resting respiratory rate, head-neck alignment, and chest expansion among university students.

Methodology

A cross-sectional observational study was conducted involving 300 university students reporting increased smartphone use. Resting respiratory rate was assessed in a relaxed sitting position. Head-neck alignment was evaluated using the craniovertebral angle (CVA), measured through lateral-view photographic analysis. Pearson's correlation analysis was performed to determine the relationships between smartphone screen time, resting respiratory rate, CVA, and chest expansion measurements. To account for multiple comparisons, a Bonferroni correction was applied, and the adjusted level of statistical significance was set at p < 0.01.

Results

Correlation analysis demonstrated a very weak negative correlation between smartphone screen time and resting respiratory rate (r = -0.0478, p = 0.4092), which was not statistically significant. A very weak positive correlation was observed between smartphone screen time and CVA (r = 0.1232, p = 0.0329); however, this association did not remain statistically significant after Bonferroni correction. A very weak negative correlation was observed between smartphone screen time and axillary chest expansion (r = -0.1574, p = 0.0063), which remained statistically significant after Bonferroni correction. The associations with fourth intercostal space chest expansion (r = -0.1139, p = 0.0488) and xiphisternal chest expansion (r = -0.0613, p = 0.2902) were not statistically significant after Bonferroni correction.

Conclusions

Smartphone screen time demonstrated predominantly weak associations with head-neck alignment and chest expansion among university students. After Bonferroni correction for multiple comparisons, only the association with axillary chest expansion remained statistically significant; however, the effect size was weak, indicating limited clinical relevance. No significant association was observed between smartphone screen time and resting respiratory rate, while the association with CVA did not remain significant after correction. Further longitudinal studies with larger sample sizes and adjustment for potential confounding factors are warranted.

## Introduction

Nowadays, smartphones have become an integral part of everyday life. Excessive smartphone use is increasingly common, with individuals spending prolonged periods engaging in activities such as social media use and gaming [1]. This may result in prolonged periods of a forward head-down posture, which can lead to several postural syndromes, such as forward head posture, rounded shoulders, and postural thoracic slouching [2]. These postural changes have become an important musculoskeletal concern due to their potential effects on physical health and activities of daily living.

Proper head and neck alignment is important for maintaining normal biomechanical balance and reducing stress on cervical structures. Forward head posture is characterized by the anterior positioning of the head relative to the trunk, leading to altered alignment of the cervical and thoracic regions. Continuous stress on the cervical musculature and surrounding soft tissues may contribute to muscular imbalance, thoracic kyphosis, and postural dysfunction [3]. If these postural alterations persist without correction, they may progress from a postural syndrome to a dysfunctional syndrome and eventually to a derangement syndrome [4]. The craniovertebral angle (CVA) is commonly used to assess head-neck alignment, and a reduced CVA is considered indicative of forward head posture.

Altered head-neck alignment may also influence respiratory mechanics and pulmonary function. Poor posture can affect thoracic expansion, diaphragmatic efficiency, and accessory muscle activity, thereby altering the normal breathing pattern [2,3]. Changes in cervical and thoracic posture may reduce chest mobility and affect respiratory function over time. As a result, resting respiratory rate and other respiratory parameters may be influenced by prolonged poor posture associated with excessive smartphone use. Therefore, assessment of respiratory alterations in individuals with altered head-neck alignment is clinically important for understanding the relationship between posture and respiratory health.

Several previous studies have investigated the relationship between smartphone use, forward head posture, and musculoskeletal disorders. Earlier research has demonstrated that prolonged smartphone use is associated with reduced CVA, neck pain, rounded shoulder posture, and postural dysfunction among students and young adults [1,5]. Some studies have also reported that poor posture may negatively affect pulmonary parameters and respiratory mechanics [2,3]. Existing literature suggests that prolonged smartphone use for more than four hours per day may contribute to postural abnormalities and reduced respiratory efficiency. A study conducted by Jung S. et al. in 2016 concluded that prolonged smartphone use of more than four hours per day was associated with a decrease in peak expiratory flow [1]. The study also showed that individuals with prolonged smartphone use tended to exhibit poorer forward head posture and rounded shoulders.

Therefore, there is a need to investigate the potential relationship between prolonged smartphone use, postural alignment, and respiratory function. Sustained smartphone screen time in a static posture may alter head and neck alignment, influence thoracic mobility, and affect the regulation of breathing, potentially leading to changes in respiratory rate. Previous studies have established an association between increased smartphone screen time and postural abnormalities. However, there is a dearth of literature regarding the correlation between smartphone screen time and resting respiratory rate. Hence, the present study aims to assess the correlation between smartphone screen time and resting respiratory rate, head-neck alignment, and chest expansion among university students. Therefore, this study was undertaken to address this gap in the existing literature.

## Materials and methods

This cross-sectional observational study was conducted at Krishna College of Physiotherapy, Krishna Vishwa Vidyapeeth, Karad, from April 2026 to June 2026. A total of 300 university medical students were recruited using convenience sampling. Data were collected using a structured data collection sheet. The sample size was determined based on the feasibility of participant recruitment during the study period and in consultation with the research supervisor. Smartphone screen time was obtained from the device's built-in screen-time report.

Head-neck alignment was assessed using the CVA, measured from standardized lateral-view digital photographs of each participant captured using a mobile phone camera while standing in a relaxed posture. The camera was positioned at a standardized height and distance for all participants. The craniovertebral angle was defined as the angle formed by a horizontal line passing through the spinous process of the seventh cervical vertebra (C7) and a line connecting C7 to the tragus of the ear. The angle was measured from the digital photographs. No radiographic or other imaging procedures were performed. According to the conventional definition, a smaller craniovertebral angle indicates a greater degree of forward head posture, whereas a larger craniovertebral angle indicates a more upright head posture.

Resting respiratory rate was recorded using a stopwatch while the participant was seated comfortably and breathing normally. Chest expansion was measured using a measuring tape at three anatomical levels: the axillary level, the fourth intercostal space, and the xiphisternum level. Axillary level expansion was defined as the difference in chest circumference measured at the axillary fold during maximal inspiration and expiration. Fourth intercostal space expansion was defined as the difference in chest circumference measured at the level of the fourth intercostal space during maximal inspiration and expiration. Xiphisternum level expansion was defined as the difference in chest circumference measured at the level of the xiphisternum during maximal inspiration and expiration.

The outcome measures included smartphone screen time, resting respiratory rate, craniovertebral angle, and chest expansion. Statistical analysis was performed using GraphPad InStat version 3.10 (GraphPad Software, San Diego, CA, USA). Pearson's correlation coefficient (r) was used to assess the association between smartphone screen time and resting respiratory rate, craniovertebral angle, and chest expansion measurements. A two-tailed p-value of < 0.05 was considered statistically significant. To account for multiple correlation analyses, a Bonferroni correction was applied, and the adjusted level of statistical significance was set at p < 0.01 (0.05/5).

Eligibility criteria

Inclusion Criteria

Participants aged 18-25 years with smartphone usage of more than four hours per day who were willing to participate in the study were included. Both male and female participants were recruited.

Exclusion Criteria

Individuals with cardiovascular disease, respiratory tract infections, acute neck pain, current use of medications, those undergoing physiotherapy sessions, individuals with a history of smoking or tobacco use, and individuals with known musculoskeletal, neurological, congenital, or postural disorders affecting head-neck alignment were excluded from the study.

Ethical approval

The study was approved by the Institutional Ethics Committee of Krishna Vishwa Vidyapeeth (Deemed to be University), Karad, Maharashtra, India (Approval No. 537/2025-2026). After obtaining ethical approval, eligible participants were informed about the study, and written informed consent was obtained from all participants before enrollment.

**Figure 1:**
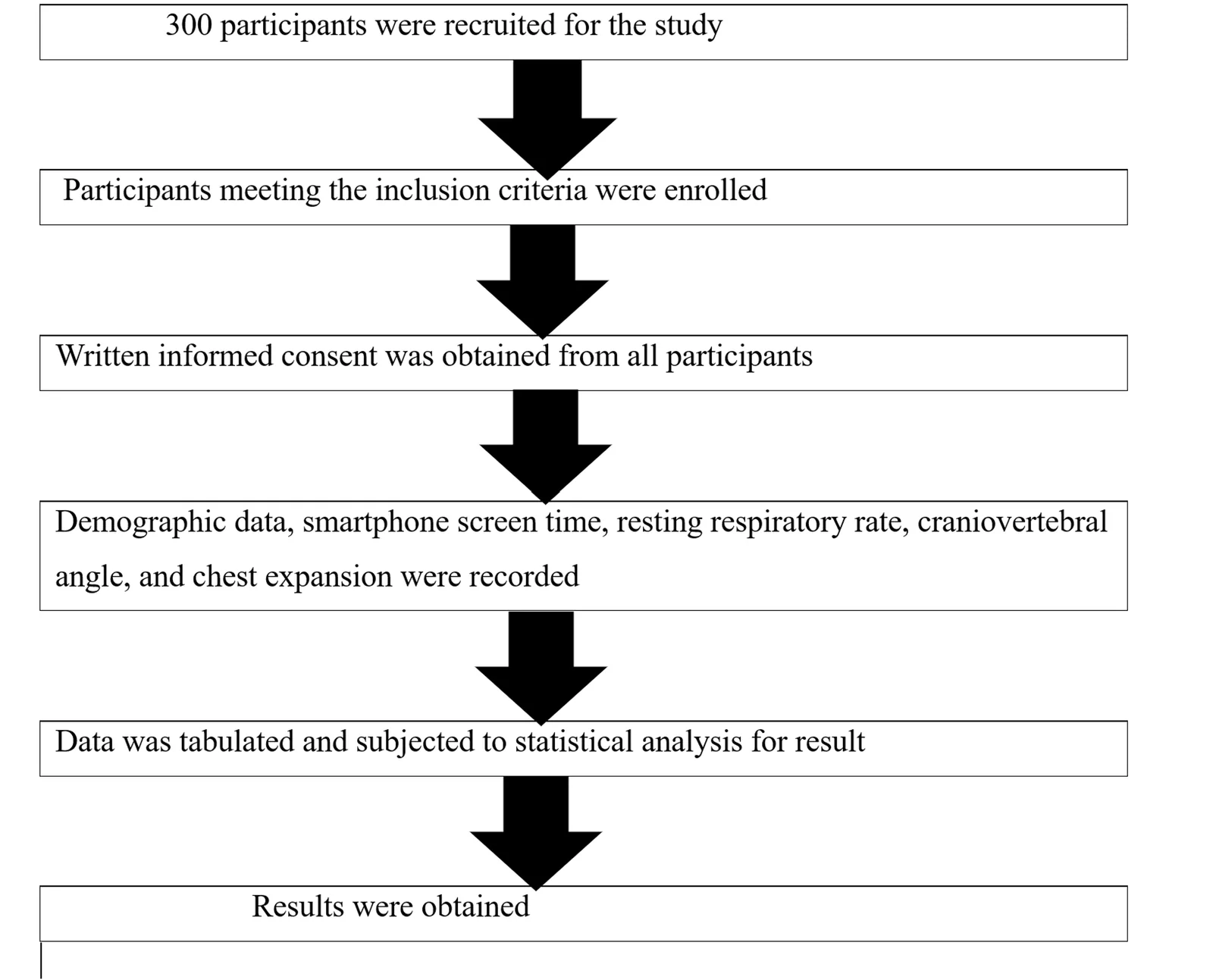
Flow diagram illustrating participant recruitment, screening, data collection, and statistical analysis procedures

The participant recruitment, screening, and study procedure are illustrated in Figure 1.

## Results

The demographic characteristics of the participants are presented in Table 1. A total of 300 university students participated in the study. The mean age of the participants was 21.35 ± 1.31 years. Female participants constituted the majority of the sample (79.0%), while males accounted for 21.0%. The mean height, weight, and BMI of the participants were 162.52 ± 9.82 cm, 61.53 ± 11.72 kg, and 23.34 ± 4.58 kg/m², respectively.

**Table 1:** Demographic characteristics of the participants (N = 300) SD: standard deviation; BMI: body mass index

Variable	Value
Age, years, mean ± SD	21.35 ± 1.31
Gender - male, n (%)	63 (21.0%)
Gender - female, n (%)	237 (79.0%)
Height, cm, mean ± SD	162.52 ± 9.82
Weight, kg, mean ± SD	61.53 ± 11.72
BMI, kg\m^2^, mean ± SD	23.34 ± 4.58

Table 2 presents the descriptive statistics of the study outcome measures, including smartphone screen time, resting respiratory rate, chest expansion measurements at different levels, and craniovertebral angle among the participants. The mean smartphone screen time was 5.51 ± 1.01 hours/day, while the mean resting respiratory rate was 17.52 ± 2.87 breaths/min. The mean chest expansion was 2.10 ± 0.83 cm at the axillary level, 1.83 ± 0.92 cm at the 4th intercostal space, and 1.90 ± 0.84 cm at the xiphisternal level. The mean craniovertebral angle was 43.17 ± 4.08 degrees.

**Table 2:** Descriptive statistics of outcome measures (N = 300) SD: standard deviation

Variable	Mean ± SD
Smartphone screen time, hours/day	5.51 ± 1.01
Resting respiratory rate, breaths/min	17.52 ± 2.87
Chest expansion at axillary level, cm	2.10 ± 0.83
Chest expansion at 4th intercostal space, cm	1.83 ± 0.92
Chest expansion at xiphisternal level, cm	1.90 ± 0.84
Craniovertebral angle, degrees	43.17 ± 4.08

Table 3 shows the correlation between smartphone screen time and respiratory rate. A very weak negative correlation was observed between smartphone screen time and respiratory rate (r = -0.0478). However, this association was not statistically significant (p = 0.4092), suggesting that smartphone screen time did not have a significant relationship with respiratory rate in the study population.

**Table 3:** Correlation between smartphone screen time and respiratory rate Pearson's correlation test

Variable	Correlation coefficient (r)	P-value
Screen time vs. respiratory rate	-0.0478	0.4092

Table 4 shows the correlation between smartphone screen time and craniovertebral angle. A very weak positive correlation was observed (r = 0.1232), and the relationship was statistically significant at the conventional level (p = 0.0329); however, it did not remain statistically significant after Bonferroni correction (adjusted significance level, p < 0.01).

**Table 4:** Correlation between smartphone screen time and craniovertebral angle Pearson's correlation test

Variable	Correlation coefficient (r)	P-value
Screen time vs. craniovertebral angle	0.1232	0.0329

Table 5 shows the correlation between smartphone screen time and axillary chest expansion. Correlation analysis revealed a very weak negative correlation between smartphone screen time and axillary chest expansion (r = -0.1574, p = 0.0063). This association remained statistically significant after Bonferroni correction (adjusted significance level, p < 0.01). However, the correlation was weak, indicating limited clinical significance.

**Table 5:** Correlation between smartphone screen time and axillary-level expansion Pearson's correlation test

Variable	Correlation coefficient (r)	P-value
Screen time vs. axillary-level expansion	-0.1574	0.0063

Table 6 shows the correlation between smartphone screen time and 4th intercostal space chest expansion. Correlation analysis revealed a very weak negative correlation between smartphone screen time and fourth intercostal chest expansion (r = -0.1139, p = 0.0488). Although statistically significant at the conventional significance level (p < 0.05), this association did not remain statistically significant after Bonferroni correction.

**Table 6:** Correlation between smartphone screen time and 4th intercostal space expansion Pearson's correlation test

Variable	Correlation coefficient (r)	P-value
Screen time vs. 4th intercostal space expansion	-0.1139	0.0488

Table 7 shows the correlation between smartphone screen time and xiphisternum level chest expansion. A very weak negative correlation was observed between smartphone screen time and xiphisternum level chest expansion (r = -0.0613). However, this association was not statistically significant (p = 0.2902), indicating that smartphone screen time was not significantly related to xiphisternum level chest expansion among the study participants.

**Table 7:** Correlation between smartphone screen time and xiphisternal-level expansion Pearson's correlation test

Variable	Correlation coefficient (r)	P-value
Screen time vs. xiphisternal-level expansion	-0.0613	0.2902

## Discussion

The present study investigated the relationship between smartphone usage and selected postural and respiratory parameters in young adults. The findings demonstrated no statistically significant association between smartphone screen time and resting respiratory rate (r = -0.0478, p = 0.4092). A weak positive correlation was observed between smartphone screen time and CVA (r = 0.1232, p = 0.0329). Although this association reached the conventional significance level of p < 0.05, it did not remain statistically significant after Bonferroni correction (adjusted significance level p < 0.01). A very weak negative correlation was observed between smartphone screen time and axillary chest expansion (r = -0.1574, p = 0.0063), which remained statistically significant after Bonferroni correction. The correlations with fourth-intercostal chest expansion (r = -0.1139, p = 0.0488) and xiphisternal chest expansion (r = -0.0613, p = 0.2902) did not remain statistically significant after correction. Overall, the observed correlations were weak, suggesting limited clinical significance.

Previous literature has reported associations between prolonged smartphone use and forward head posture. Jung et al. [1] reported that smartphone use exceeding four hours per day is associated with forward head posture and reduced pulmonary function, highlighting the potential postural impact of prolonged device use. Similarly, Lee and Song [6] found a significant relationship between smartphone usage and forward head posture in healthy adults. In the present study, however, the association between smartphone screen time and CVA did not remain statistically significant after Bonferroni correction. Therefore, although the present finding is directionally related to previous reports of postural changes associated with smartphone use, it should be interpreted cautiously.

According to the conventional definition of CVA, a smaller angle indicates a greater degree of forward head posture, whereas a larger angle reflects a more upright head-neck alignment. In the present study, a weak positive correlation was observed between smartphone screen time and CVA (r = 0.1232, p = 0.0329). Although the association was statistically significant at the conventional significance level of p < 0.05, it did not remain statistically significant after Bonferroni correction (adjusted significance level p < 0.01). Furthermore, the direction of this association differs from that commonly reported in the literature, where prolonged smartphone use is generally associated with a reduced CVA and greater forward head posture. Differences in measurement techniques, participant characteristics, smartphone usage behavior, ergonomic factors, and other unmeasured confounding variables may have influenced the observed findings. Therefore, further studies are needed to clarify the direction and clinical significance of the relationship between smartphone screen time and head-neck alignment.

Several factors other than smartphone screen time may influence head-neck alignment and CVA. These include prolonged use of laptops and desktop computers, studying posture, reading habits, physical activity levels, ergonomic factors, backpack use, academic workload, and individual musculoskeletal characteristics. As these potential confounding factors were not specifically controlled for in the present study, they may have contributed to the observed variations in head-neck posture. Therefore, the association between smartphone screen time and CVA should be interpreted with caution.

The present findings are further supported by previous studies investigating the effects of smartphone use on posture and respiratory function. Kang et al. [2] reported that sitting posture while using a smartphone can influence respiratory function, suggesting that prolonged device use may contribute to postural adaptations that affect breathing mechanics. Similarly, Koseki et al. [3] demonstrated that forward head posture alters thoracic shape and respiratory function, highlighting the biomechanical relationship between cervical alignment and respiratory performance. Faisal et al. [4] also observed significant associations between smartphone usage, CVA, and pulmonary function, supporting the relationship between prolonged smartphone use and postural alterations observed in the present study. Furthermore, Saeed et al. [5] reported a high prevalence of forward head posture among university students and identified a significant association between smartphone use and postural deviations. These findings provide a broader context for the potential relationship between smartphone use and head-neck alignment; however, the present study did not demonstrate a statistically significant association between smartphone screen time and CVA after Bonferroni correction.

Furthermore, Kim et al. [7] identified correlations between forward head posture, respiratory function, and respiratory accessory muscle activity in young adults, indicating that cervical alignment plays a role in respiratory mechanics. Kang et al. [8] also reported similar associations between forward head posture and pulmonary function impairment, emphasizing the biomechanical link between posture and respiratory performance. The present findings are also supported by Lee NK et al. [9], who demonstrated that exercise interventions can improve cervical angle and respiratory function in smartphone users, indirectly suggesting that altered posture due to smartphone use may negatively influence musculoskeletal and respiratory efficiency. In addition, Kim [10] reported that prolonged smartphone use influences cervical movement patterns in the sagittal plane, further reinforcing the mechanical stress placed on the cervical spine during sustained device usage.

Despite these findings, the present study did not observe a significant relationship between smartphone screen time and resting respiratory rate. The association between smartphone screen time and axillary chest expansion remained statistically significant after Bonferroni correction; however, the correlation was very weak (r = -0.1574), suggesting limited clinical significance. The associations with fourth-intercostal and xiphisternal chest expansion were not statistically significant after correction. The weak correlations observed for most respiratory parameters may indicate that any relationship between smartphone usage and respiratory function is small. These findings should be interpreted with caution and may reflect the ability of healthy individuals to maintain normal respiratory function despite variations in smartphone usage patterns. This contrasts with earlier studies that reported associations between forward head posture and pulmonary function parameters. However, most of those studies assessed dynamic pulmonary measures such as forced vital capacity, peak expiratory flow rate, and respiratory muscle activity rather than resting respiratory rate. Therefore, the lack of a significant association between smartphone screen time and resting respiratory rate may be attributed partly to the limited sensitivity of resting respiratory rate as a physiological marker and the compensatory respiratory mechanisms present in healthy young adults.

The weak negative correlations observed between smartphone screen time and chest expansion may also be explained by the early stage of postural adaptation in the studied population. The association with axillary chest expansion remained statistically significant after Bonferroni correction; however, the correlation was weak, indicating that the observed relationship was small. The fourth-intercostal and xiphisternal measurements did not show statistically significant associations after correction. Structural respiratory changes may be more likely to appear with prolonged postural dysfunction rather than short-term or moderate exposure. Additionally, chest expansion may remain within normal compensatory limits despite early cervical postural alterations.

The strengths of this study include a relatively large sample size (n = 300), standardized measurement techniques for CVA and respiratory parameters, and a focus on an emerging lifestyle-related health concern in young adults. However, the cross-sectional design limits causal inference. Additionally, restricting the sample to university students limits generalizability to other populations. The use of resting respiratory rate and chest expansion alone, without comprehensive pulmonary function testing, may also limit the sensitivity of respiratory assessment.

Future research should employ longitudinal or interventional designs to better establish causality between smartphone usage and postural-respiratory outcomes. Inclusion of detailed pulmonary function tests such as forced vital capacity, forced expiratory volume, and peak expiratory flow rate would provide a more comprehensive understanding of respiratory involvement. Studies involving broader age groups and occupational populations are also recommended to improve external validity.

A limitation of the present study is that potential confounding factors such as physical activity level, neck pain, occupation, BMI, and other lifestyle-related factors were not assessed or adjusted for in the statistical analysis. Therefore, the observed associations should be interpreted with caution, as these variables may have influenced the study findings. Future studies should include these potential confounders and apply multivariable statistical analyses to better understand the independent relationship between smartphone screen time, head-neck alignment, and resting respiratory rate. In addition, the present study did not assess participants' history of previous neck or cervical trauma, occupational activities, or other daily activities that may influence head-neck posture. These factors could have affected the CVA measurements and should be considered in future studies.

## Conclusions

The present study demonstrated predominantly weak associations between smartphone screen time and postural and respiratory parameters among university students. After Bonferroni correction for multiple comparisons, only the association with axillary chest expansion remained statistically significant, although the observed effect size was small, indicating limited clinical significance. No significant association was observed between smartphone screen time and resting respiratory rate, while the association with CVA did not remain significant after correction. Further longitudinal studies with larger sample sizes and appropriate adjustment for potential confounding factors are recommended.
